# Formation of xenon-nitrogen compounds at high pressure

**DOI:** 10.1038/srep34896

**Published:** 2016-10-17

**Authors:** Ross T. Howie, Robin Turnbull, Jack Binns, Mungo Frost, Philip Dalladay-Simpson, Eugene Gregoryanz

**Affiliations:** 1 Center for High Pressure Science & Technology Advanced Research, Shanghai, 201203, P.R. China; 2 Centre for Science at Extreme Conditions and School of Physics and Astronomy, University of Edinburgh, Edinburgh, UK

## Abstract

Molecular nitrogen exhibits one of the strongest known interatomic bonds, while xenon possesses a closed-shell electronic structure: a direct consequence of which renders both chemically unreactive. Through a series of optical spectroscopy and x-ray diffraction experiments, we demonstrate the formation of a novel van der Waals compound formed from binary Xe-N_2_ mixtures at pressures as low as 5 GPa. At 300 K and 5 GPa Xe(N_2_)_2_-I is synthesised, and if further compressed, undergoes a transition to a tetragonal Xe(N_2_)_2_-II phase at 14 GPa; this phase appears to be unexpectedly stable at least up to 180 GPa even after heating to above 2000 K. Raman spectroscopy measurements indicate a distinct weakening of the intramolecular bond of the nitrogen molecule above 60 GPa, while transmission measurements in the visible and mid-infrared regime suggest the metallisation of the compound at ~100 GPa.

Nitrogen is the most abundant element in the terrestrial atmosphere, existing as a diatomic molecule with one of the strongest known triple bonds and as a result is unreactive at ambient conditions. Under high compression, molecular nitrogen exhibits a rich polymorphism^1^,^2^,^3^,^4^,^5^,^6^,^7^ and significant overlap of thermodynamically competing phases, dependent on formation conditions^8^. The application of high pressure can also provide new synthesis routes, initiating chemical processes that would not happen otherwise, such as N_2_ becoming reactive with the noble metals, as in the formation of platinum or iridium nitrides^9^,^10^. Xenon, an archetypical inert gas due to its closed shell system, has long been known to form stable halide and oxide compounds through chemical synthesis^11^,^12^. The reactivity can also be fundamentally altered with the application of high pressure, the process which has produced van der Waals compounds composed of Xe-H_2_^13^ and Xe-O_2_^14^, as well as a Xe-H_2_O clathrate^15^. Direct reactions have also been observed such as that between xenon and ice^16^ and the recently reported stable oxides, Xe_2_O_5_ and Xe_3_O_2_^17^. Xenon has also been shown to be inserted into both quartz^18^, and a small-pore zeolite at high pressure and temperature^19^. Theoretical studies also suggest the increased reactivity of xenon at high pressures with the formation of binary solids Xe-O^20^,^21^, Xe-Fe/Ni^22^, and Xe-Mg^23^ synthesised solely from their constituent elements. Such studies on the reactivity of xenon, especially with terrestrially abundant elements, could provide an explanation into the significant under-abundance of xenon detectable in the Earth’s atmosphere.

The direct reaction of N_2_ and Xe would seem unlikely due to the relative inertness of both materials. Nevertheless, a recent theoretical study predicts the formation of novel xenon nitride compounds above 146 GPa with stoichiometry - XeN_6_^24^. Possible interactions between Xe and N_2_ have been explored experimentally at low pressures investigating mutual solubility^25^,^26^. Through Raman spectroscopic measurements those studies inferred the formation of an orientationally disordered van der Waals compound but were limited up to pressures of 13 GPa at 408 K with no structural investigation.

It is known that at high pressures both xenon and nitrogen exhibit (semi-)conducting phases. Xenon has been shown to transform to metallic state at pressures between 130–150 GPa, giving it the lowest pressure of metallisation amongst the rare gas solids^27^,^28^,^29^ and nitrogen becomes semiconducting with band gap of 0.4 eV at 240 GPa^2^,^3^. Previous studies have claimed that by doping Xe with O_2_, the metallisation pressure is drastically reduced^30^. Therefore it is of significant interest to investigate pressure-induced electronic effects of any formed Xe-N_2_ compound. Here, we report the synthesis and characterisation of a Xe-N_2_ van der Waals compound through x-ray diffraction, Raman and transmission spectroscopies. We show that two inert condensed gases form a Xe(N_2_)_2_ compound at pressures as low as 5 GPa at room temperature. When the novel compound is formed in a xenon medium, it becomes metallic at around 100 GPa, whilst Xe(N_2_)_2_ with an abundance of nitrogen demonstrates metallic behaviour above ~140 GPa.

Mixtures of Xe-N_2_ at various concentration were loaded into diamond-anvil cells (DAC) using a combination of cryogenic and high-pressure gas-loading techniques (see Methods section). Compressing the mixture above 2 GPa leads to the formation of a xenon single crystal surrounded by liquid N_2_ as seen visually and in x-ray diffraction measurements (see Figs S1 and S2). At pressures above 5 GPa we observe the formation of a N_2_-rich compound in the media surrounding the xenon single crystal (Fig. S2). Through x-ray powder diffraction analysis we have identified this phase as having a *fcc* structure, with *a* = 9.2361(3) Å at 5.6 GPa (Fig. 1), indexing with space group 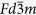 or 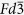 accounts for all observed Bragg peaks. Several patterns were of sufficient quality to allow for Rietveld refinement, otherwise Le Bail fitting was used to extract unit-cell dimensions. Solution of the structure by charge-flipping suggests space group 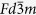. Two atomic sites could be refined; Xe(0, 0, 0) and N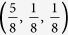 resulting in a cubic Laves Cu_2_Mg-type structure (Fig. 1(a)).

From both the structure type and unit-cell dimensions we determine the stoichiometry as Xe(N_2_)_2_, designated Xe(N_2_)_2_-I herein, which is in excellent agreement with the calculated equation-of-state data for Xe + 4N (Fig. 1(b), see also below). Both the structure type and the stoichiometry are identical to that proposed for oxygen-rich xenon mixtures^14^. The N-N site distances of 3.2655(1) Å are clearly too long to be bonded, these sites therefore represent scattering from disordered N_2_ molecules. N_2_ molecules have been found to adopt both spherical and disk-like rotational disordering in the solid state^31^, and refinement of both disorder types was attempted, with a spherical disorder model (*i.e.* with the N-site occupancy equal to 2) resulting in the best fit to the data (see table in SM for more details on the structure refinement). The structure of this phase can be considered as a diamond-type host lattice of Xe atoms with four rotationally disordered N_2_ molecules forming a tetrahedron within each vacancy. The N-N site distance of 3.2655(1) Å implies a N…N closest-contact distance of 2.1655(1) Å.

Raman spectroscopy measurements of the formed single crystal at 2 GPa reveals the appearance of a weak vibrational mode, which is lower in frequency than the fluid N_2_ vibrational mode by 10 cm^−1^ (compare red and black spectra in Fig. 2). This mode has been observed in a previous high-temperature study and attributed to fluid N_2_ dissolved in the Xe crystal lattice^26^. By contrast, in xenon-rich samples (*ca.* 4:1 concentration), we observe the complete transformation of the sample, evident through only the low-frequency vibrational mode and no evidence of excess N_2_ (see SM).

In Raman measurements of the surrounding media (see blue spectra in Fig. 2), we observe a broad N_2_ mode at 5 GPa, which consists of overlapping modes of Xe(N_2_)_2_-I, as determined by x-ray diffraction, and pure N_2_ that increasingly separate in frequency space at higher pressure. The vibrational mode of Xe(N_2_)_2_-I (blue spectra in Fig. 2) and the vibrational mode attributed to N_2_ in Xe (red spectra in Fig. 2), exhibit identical behaviour with pressure (see red and blue points in Fig. 3a), suggesting that the latter is most likely due to small crystallites of Xe(N_2_)_2_-I that form within a Xe matrix. It should be noted that the prescence of this N-containing dopant does not significantly affect the measured unit-cell volume which agrees with the literature to within experimental error^32^.

Above pressures of 14 GPa, we observe a phase transition from the low-pressure Xe(N_2_)_2_-I to a high-pressure Xe(N_2_)_2_-II phase. This transition pressure corresponds approximately to the critical pressure of the *δ* to 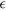 transition in pure molecular N_2_. Xe(N_2_)_2_-II adopts a body-centered tetragonal cell with *a* = 5.7228(3), *c* = 9.2134(10) Å at 18.7 GPa (Fig. 1). Systematic-absence analysis unambiguously confirm space-group symmetry *I*4_1_/*amd*. Again Xe is located at the origin, with one N position refined to (0.5, 0.721(2), 0.179(1)). This position lies displaced by 0.52(2) Å from an inversion centre resulting in four ordered N_2_ molecules aligned along the *c*-axis. Final Rietveld agreement factors are *R*_*p*_ = 0.015 and *R* = 0.094.

The origin of this transition lies in the ordered orientation of N_2_ molecules within the vacancy, corroborated by the poorer fit to the data (*R* = 0.1672) with a spherically-disordered N_2_ molecule model. Shortest N…N interatomic distances are now 2.5238(1) Å and 2.610(12) Å at 18.7 GPa. Recalling that the shortest N…N interatomic distances at 5.6 GPa were 2.1655 (1) Å in phase I, the alignment of N_2_ molecules relieves unfavourable close N…N contacts while maintaing the same coordination number for each N_2_ molecule.

Over the I-II phase transition the unit cell undergoes a tetragonal distortion elongating by 0.323(3) Å (+3.6%) along **c** accompanied by a reduction of −0.519(1) Å (−8.1%) along tetragonal **a**, corresponding to 〈110〉 in phase I (see table in SM for more details on the structure refinement). We tracked unit-cell dimensions for Xe(N_2_)_2_-II up to 58 GPa (Fig. 1(c)), confirming again the stoichiometry of the compound (Fig. 1(a)) and allowing the determination of equation-of-state parameters for both Xe(N_2_)_2_ phases (see methods section). At pressures of 38 GPa and above there were clear signs of the incipient high-pressure *hcp* phase of Xe accompanied by strong diffuse scattering and increased background at *d*-spacings overlapping with a significant number of Xe(N_2_)_2_ reflections and above 58 GPa unit-cell dimensions could not be reliably extracted from the data. However the low-angle (101) reflection could be observed up to 103 GPa (see Fig. S4).

Above 40 GPa, the frequency dependence with pressure of the vibrational mode of Xe(N_2_)_2_ deviates greatly from that of pure N_2_ (Fig. 3). The maximum in the vibrational frequency *vs.* pressure is shifted from 80 GPa in pure N_2_ to 30 GPa. In the sample in Xe matrix, we observe splitting of the vibrational band (see Fig. 2) up to 70 GPa, after which the splitting is not distinguishable due to the enhanced broadening of the modes. At 140 GPa the N_2_ vibrational frequencies of Xe(N_2_)_2_ are 2161 cm^−1^ and 2212 cm^−1^, considerably lower frequencies than either those of *κ*-N_2_ (2376 cm^−1^) or *λ*-N_2_ (2320 cm^−1^, 2400 cm^−1^). Interestingly, at 178 GPa, we observe the persistence of molecular nitrogen, which is above the pressure at which pure N_2_ is claimed to become non-molecular (*η*-N_2_)^1^,^2^,^3^. We note that although we observe a much softer N_2_ molecular mode than that just before *ζ* transforms to the non-molecular amorphous *η* phase in pure N_2_, there is no evidence that the N_2_ molecules in Xe(N_2_)_2_ dissociate to form Xe-N bonding. However, there is a clear reduction in intensity (see Fig. 2) together with a marked increase in the FWHH (see Fig. S5) indicating that the molecular N-N bond is weakening. Up to highest pressure studied (180 GPa) we see no evidence of Xe-N bonded compounds predicted by theory^24^. In an attempt to promote synthesis of such compounds, we performed laser heating of the sample to temperatures of 3000 K at 120, 150, 160 and 180 GPa but no transition was observed in either Raman spectroscopy or x-ray diffraction. It is remarkable that a van der Waals solid, the components of which are inert materials, can remain stable to such extreme conditions.

Figure 3(b) shows the transmission spectra collected from two samples with different initial ratio of Xe and N_2_. The spectra were collected with both visible and mid infrared light sources which allow the coverage of energy region between ~3 to 0.6 eV. The samples in a Xe matrix (black), appear to exhibit metallic behaviour evident by the sharp rise in the absorption in the near-IR, which shifts with pressure. By 120 GPa, no detectable transmission was observed in the visible, the sample appearance became shiny and reflected red laser light (see photomicrographs in Fig. S6). Samples of Xe(N_2_)_2_ with higher N_2_ concentrations do exhibit absorption (Fig. 3 green) but not to the same extent as in the Xe matrix, which could be due to the excess of N_2_. Pure xenon has been shown to be conductive at above 135 GPa through both absorption/reflectivity and electrical measurements^27^,^28^,^29^. The mechanism of conductivity is an indirect overlap of the 5*p* valence and 5*d* conduction bands. Although determining the mechanism was beyond the scope of this study, our results indicate that by doping Xe with N_2_, or Xe(N_2_)_2_, we are able to tune the conductive properties of Xe and lower the pressure of metallisation.

Our results demonstrate that xenon can form compounds not only with chemically reactive gases such as hydrogen or oxygen, but also with unreactive nitrogen. That such a compound forms at low pressure, exhibits metallic properties, and stable to both high-pressure and high-temperature conditions will no doubt stimulate further research in the reactivity of xenon, an element which now appears to be substantially less inert than previously thought.

## Methods

We have studied the formation conditions and stability of xenon and nitrogen compounds up to pressures of 180 GPa in a diamond anvil cell (DAC). In total 12 DAC loadings were performed. 200 *μ*m culet flat diamonds were used for experiments under 50 GPa, while 60 *μ*m and 150 *μ*m culets were used for higher pressure experiments. In all experiments rhenium foil was used as the gasket material.

The loading of the Xe-N_2_ consisted of two stages. Solid Xe (99.9% purity) was initially cryogenically loaded into a DAC under a N_2_ atmosphere. Loading was confirmed initially through comparisons of white light transmission spectra due to the change in refractive index between the empty sample chamber and loaded sample. Thorough mapping of the sample with Raman spectroscopy was carried out to ensure no impurities were present in the sample and further confirmation was obtained through x-ray diffraction analysis.

N_2_ gas (99.9% purity) was then loaded into the cell at a pressure of 20 MPa using a high-pressure gas loading system, displacing some of the pre-existing Xe gas. The volume ratio was estimated by the phase separation of Xe and N_2_ in the fluid state. Using a combination of varying pressure and temperature, single crystals of the Xe rich mixture were grown.

We have used 514 and 647 nm as excitation wavelengths in the Raman spectroscopy measurements using a custom-built micro-focussed Raman system. Pressure was determined through both ruby fluorescence (P < 100 GPa) and the Raman edge of the stressed diamond^33^.

Powder x-ray diffraction data were collected at several beamlines: BL10XU at SPring-8 (Japan), IDB PETRA-III (Germany), ID09 at the European Synchrotron Radiation Facility (France), and ID-BMD of HPCAT at APS (USA). Incident beam energies in the range 25–30 keV were used. Intensity *vs.* 2*θ* plots were obtained by integrating image plate data in various formats using DIOPTAS^34^. Indexing was carried out in GSAS-II^35^, Le Bail and Rietveld refinements were carried out in Jana20006^36^. Equation of state data were determined using EosFIT 7^37^. Fitted equation of state parameters for Xe(N_2_)_2_-I: *V*_0_ = 873(90) Å^3^, *K*_0_ = 47(56) GPa, *K*′ = 1(5). Equation-of-state parameters for Xe(N_2_)_2_-II: *V*_0_ = 526(158) Å^3^, *K*_0_ = 9(14) GPa, *K*′ = 4.5(11), *K*″ = −0.51131 GPa^−1^.

## Additional Information

**How to cite this article**: Howie, R. T. *et al*. Formation of xenon-nitrogen compounds at high pressure. *Sci. Rep.* **6**, 34896; doi: 10.1038/srep34896 (2016).

## Supplementary Material

Supplementary Information

## Figures and Tables

**Figure 1:**
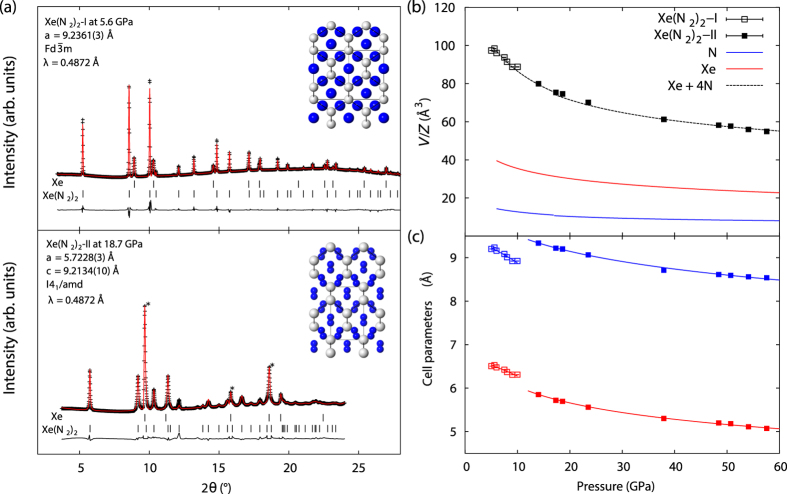
(**a**) Powder X-ray diffraction patterns at 5.6 and 18.7 GPa used for Rietveld refinement. Below 14 GPa, Xe(N_2_)_2_ adopts a face-centered cubic structure, space group 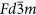, *a* = 9.2361(3) Å denoted Xe(N_2_)_2_-I. At 14 GPa and above Xe(N_2_)_2_ undergoes a transition to a body-centered tetragonal structure, *I*4_1_/*amd*, with unit-cell dimensions *a* = 5.7228(3), *c* = 9.2134(10) Å at 18.7 GPa. Peaks corresponding to Xe (marked with *) were excluded from the profile used in the refinement. Insets show crystal-structure projections for both phases, phase I is rotated to view down the face diagonal 〈110〉 highlighting structural similarity to phase II. Freely rotating N_2_ molecules in phase I are represented by blue spheres, whilst in phase II blue spheres represent atoms in aligned N_2_ molecules; (**b**) Equation-of-state data for Xe(N_2_)_2_ compounds. Pressure-volume per *Z* data for Xe phases is indicated by red lines^32^, N_2_ phases by blue lines^6^. Black squares indicate volume per Z data for Xe(N_2_)_2_ phases I and II, dashed black line indicates the calculated volume for stoichiometry Xe + 4N from atomic volume data; (**c**) Response of unit-cell dimensions to applied pressure for Xe(N_2_)_2_, phase I data are shown for unit-cell length *a* (blue open squares) and *d*_〈110〉_ (red open squares), phase II data is plotted for unit-cell lengths *a* (red closed squares) and *c* (blue closed squares). Solid lines indicate fitted linear Birch-Murnaghan linear equations of state?

**Figure 2:**
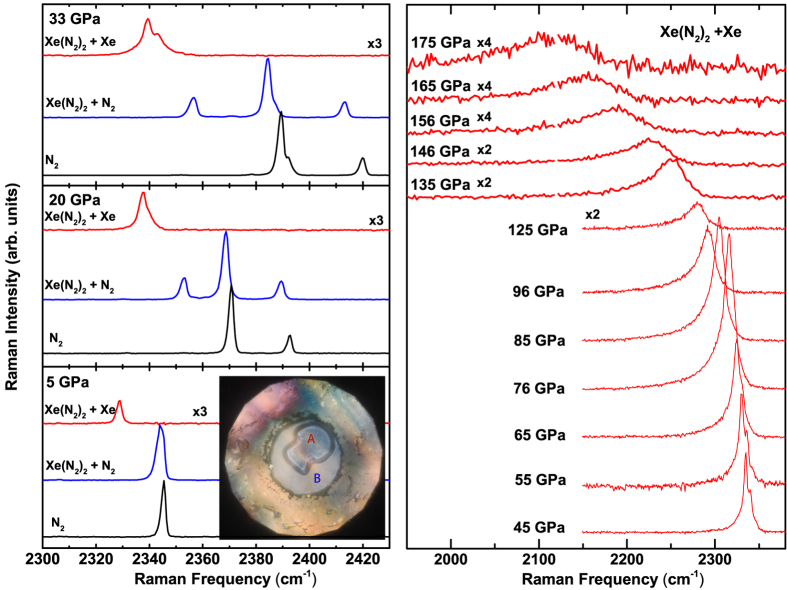
(**a**) Representative vibrational Raman spectra of the Xe-N_2_ compound at 5, 20 and 33 GPa. Red spectra are from the formed compound in Xe media, whilst blue spectra show the compound formed in N_2_ media. As a comparison, vibrational spectra of pure N_2_ are shown in black. Inset: Photomicrograph of sample at 5 GPa. Red spectra were taken at position A in the single crystal and blue spectra were taken in the surrounding medium at position B. (**b**) Representative vibrational Raman spectra of Xe(N_2_)_2_ in a Xe matrix to pressures of 175 GPa.

**Figure 3:**
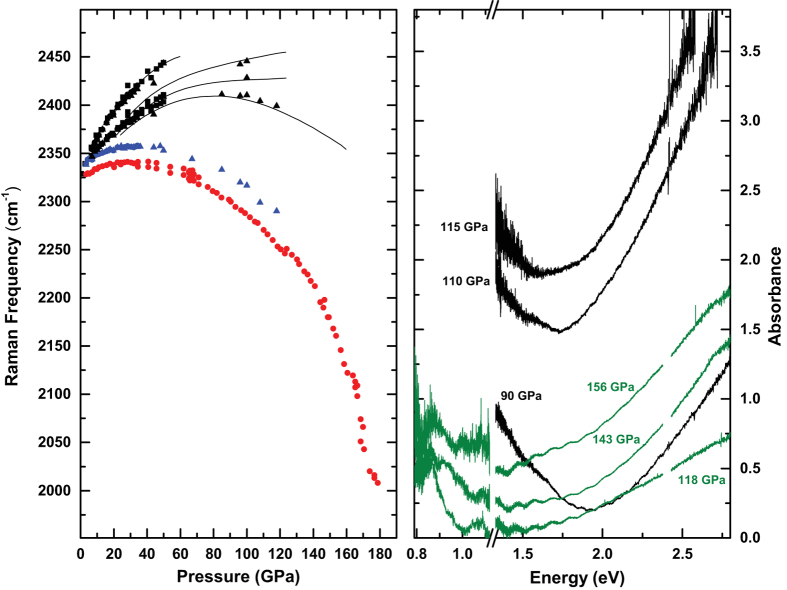
Left Panel: Frequencies of the vibrational modes as a function of pressure. Xe(N_2_)_2_ Raman frequencies in Xe medium are shown in red, Xe(N_2_)_2_ Raman frequencies in N_2_ medium are shown in blue and black points are the Raman frequencies of the excess N_2_. Black lines are taken from a study on pure N_2_^1^. Right Panel: Optical absorption as a function of energy for Xe-rich (black) and N_2_-rich (green) samples. The reference spectra were taken at 50 GPa in both experiments.
