# Supplementary material for: Formation of xenon-nitrogen compounds at high pressure
Source: Sci Rep. 2016 Oct 17;6:34896. doi: 10.1038/srep34896 (PMC5066244; doi:10.1038/srep34896)
Supplement: Supplementary Information [file srep34896-s1.pdf]

Supplementary Information

Formation of xenon-nitrogen compounds at high pressure

Ross T. Howie[1*], Robin Turnbull[2], Jack Binns[1], Mungo Frost[2], Philip Dalladay-Simpson [1], Eugene Gregoryanz[2]

[1] Center for High Pressure Science & Technology Advanced Research, Pudong, Shanghai, P.R. China.
[2] Centre for Science at Extreme Conditions and School of Physics and Astronomy, University of Edinburgh, Edinburgh EH9 3JZ, UK.

*e-mail: ross.howie@hpstar.ac.cn

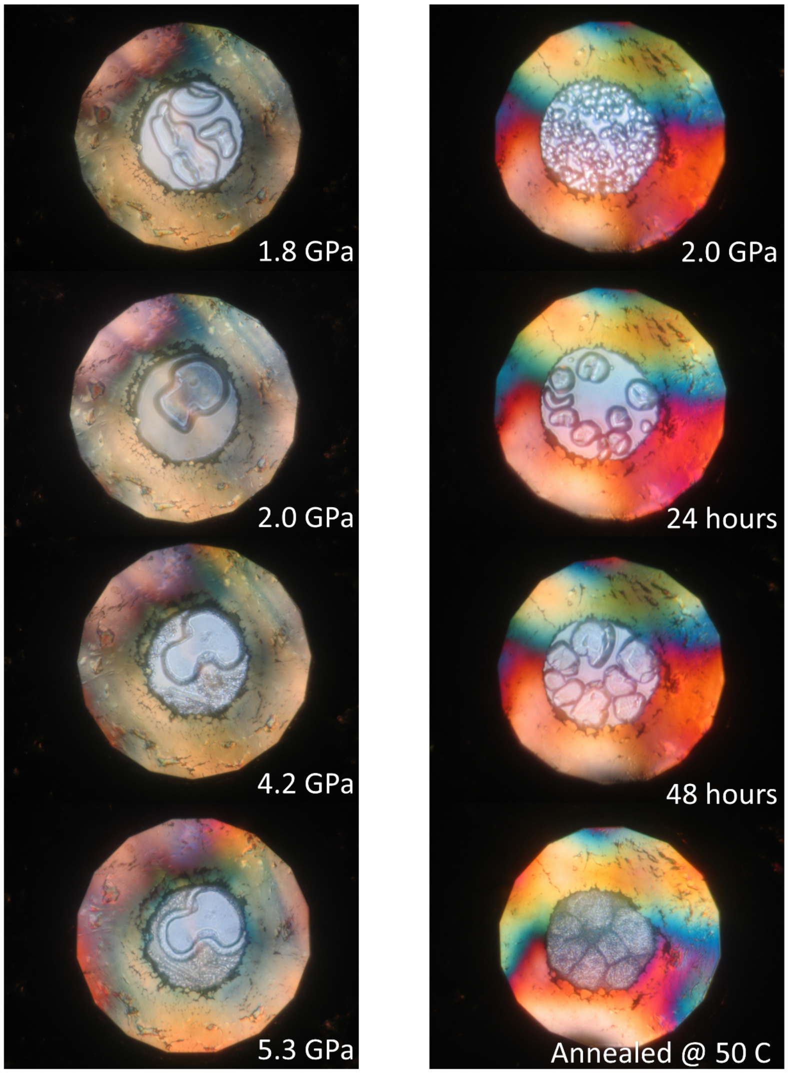

Figure S1. Micrographs of Xe-N_2 samples at low pressures. The left column shows the behaviour of a Xe-N_2 mixture of roughtly 50/50 on compression to 5.3 GPa. The ”8” shape figure is Xe single crystal embedded in a $Xe(N_2)_2$. The right column shows the evolution of a Xe-rich N_2/Xe mixture over a 48 hour period after sample loading at constant pressure.

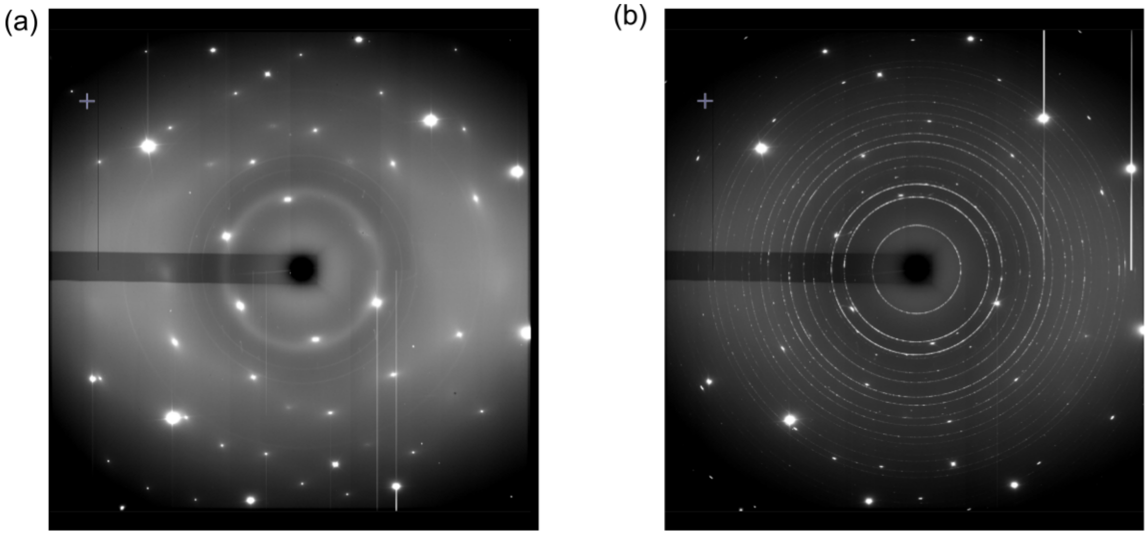

Figure S2. X-ray diffraction image plates (a) Data collected at 3 GPa shows the presence of the Xe fcc phase and the first diffraction peak from liquid nitrogen; (b) At 5.6 GPa the diffraction pattern of polycrystalline $Xe(N_2)_2$ phase dominates the image plate. Additional peaks are due to excess Xe and scattering from the diamond anvils.

	Phase I	Phase II
Crystal data		
Chemical formula	$Xe(N_2)_2$	$Xe(N_2)_2$
M_r	243.3	243.3
Crystal system, space group	Cubic, $Fd\bar{3}m$	Tetragonal, $I4_1/amd$
Pressure (GPa)	5.6	18.7
a (Å)	9.2361 (3)	5.7228 (3)
c (Å)	9.2361 (3)	9.2134 (10)
V (Å^3)	787.88 (5)	301.74 (4)
Z	8	4
Radiation type	Synchrotron ($\lambda = 0.4872$ Å)	Synchrotron ($\lambda = 0.4872$ Å)
Data collection		
2θ values (°)	$2\theta_{min} = 3.416$, $2\theta_{max} = 33.451$, $2\theta_{step} = 0.011$	$2\theta_{min} = 3.724$, $2\theta_{max} = 24.979$, $2\theta_{step} = 0.011$
Refinement		
R factors and goodness of fit	$R_p = 0.009$, $R_{wp} = 0.019$, $R_{exp} = 0.018$, $R(F) = 0.082$, $\chi^2 = 1.103$	$R_p = 0.015$, $R_{wp} = 0.021$, $R_{exp} = 0.018$, $R(F) = 0.094$, $\chi^2 = 1.369$
No. of parameters	8	17

TABLE I. Crystal structure data, experiment and refinement details for $Xe(N_2)_2$ phases I and II.

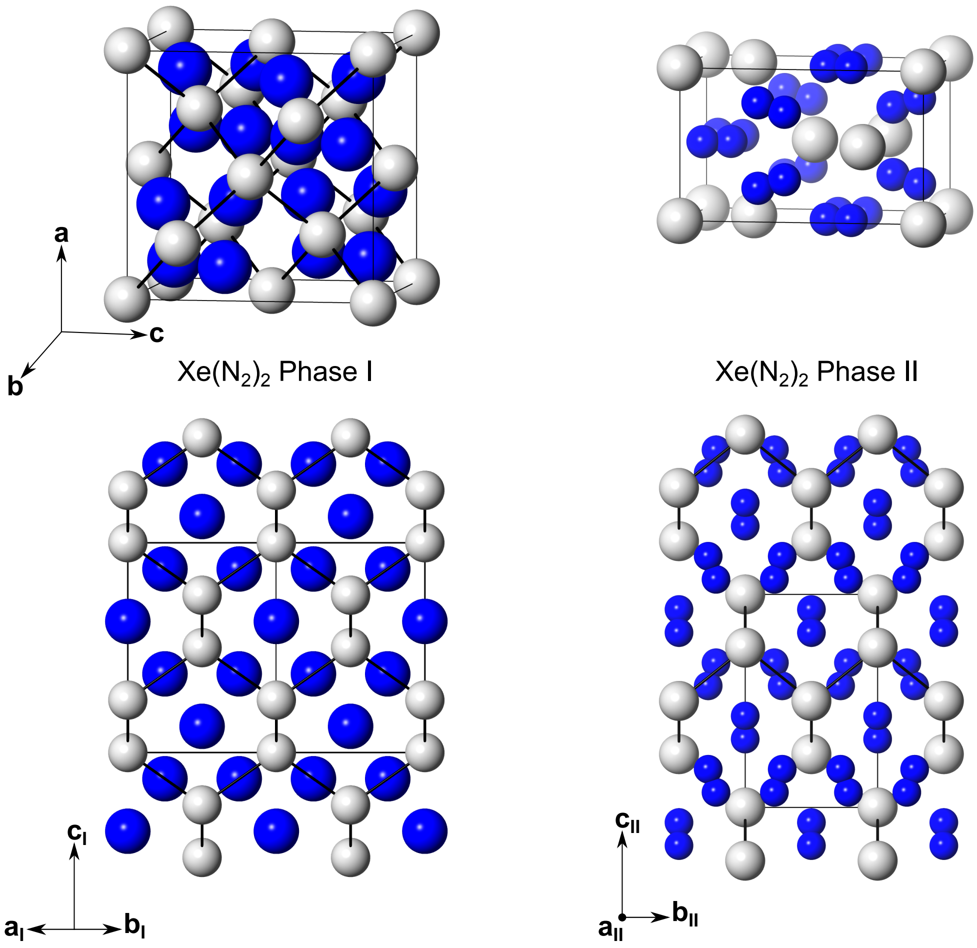

Figure S3. Crystal structures of $Xe(N_2)_2$ phases I and II. Ordering and alignment of N_2 molecules occurs over the transition leading to a tetragonal distortion along $\mathbf{c}_{II}$. Freely rotating N_2 molecules in phase I are represented by blue balls, whilst in phase II blue balls represent atoms in aligned molecules.

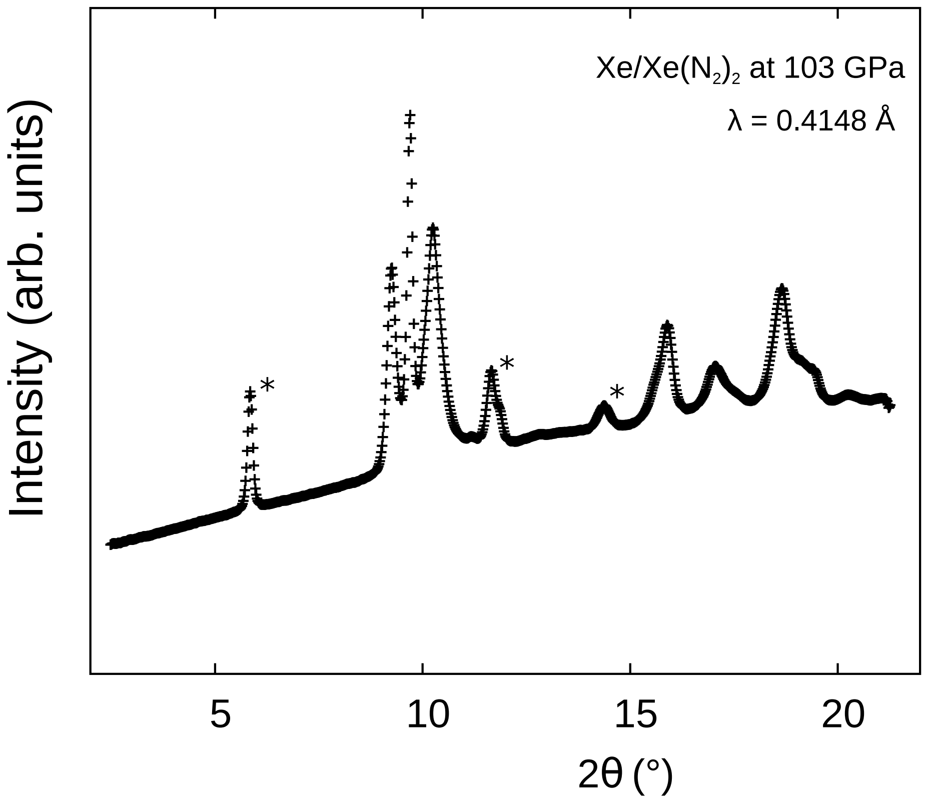

Figure S4. X-ray diffraction pattern of $Xe/Xe(N_2)_2$ at 103 GPa. Low-angle peak marked by * corresponds to the (101) peak of $Xe(N_2)_2$, additional diffracted intensity marked by * are also due to $Xe(N_2)_2$, although an unambiguous indexing is not possible due.

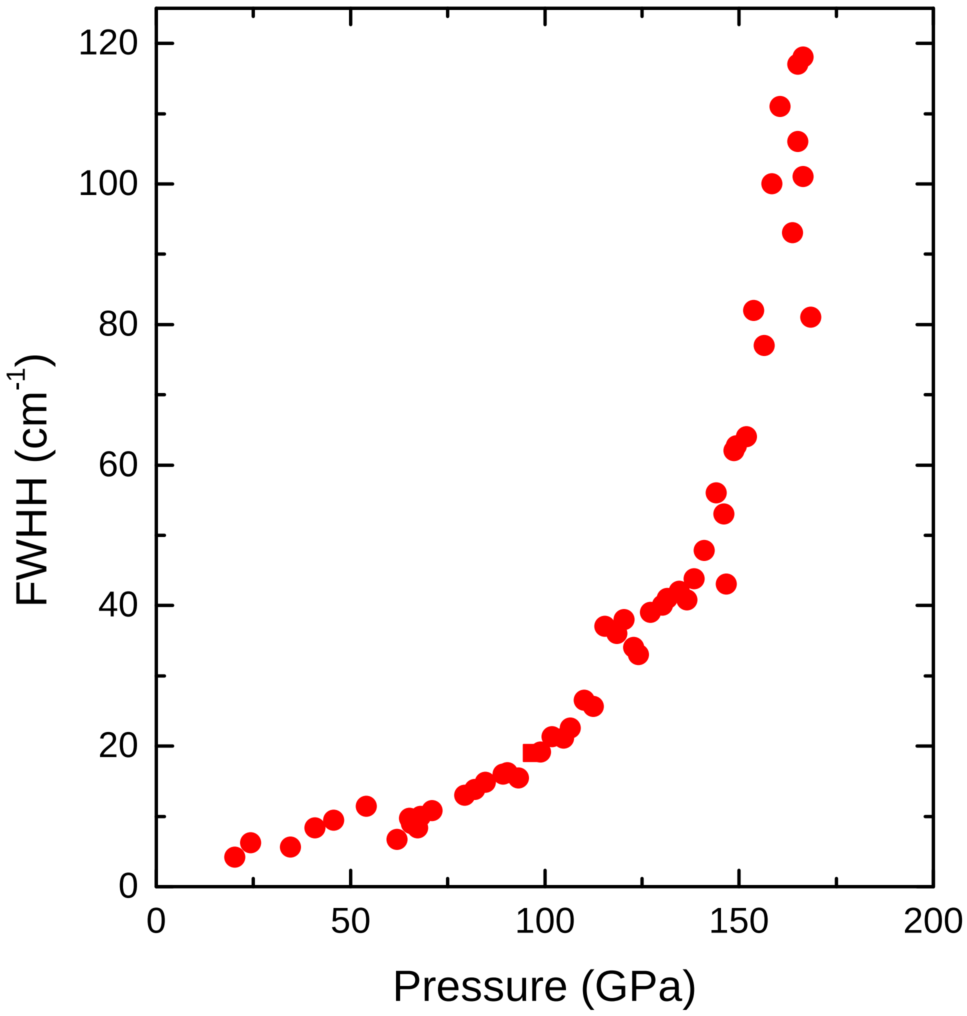

Figure S5. Full width at half height as a function of pressure for the Xe-rich sample.

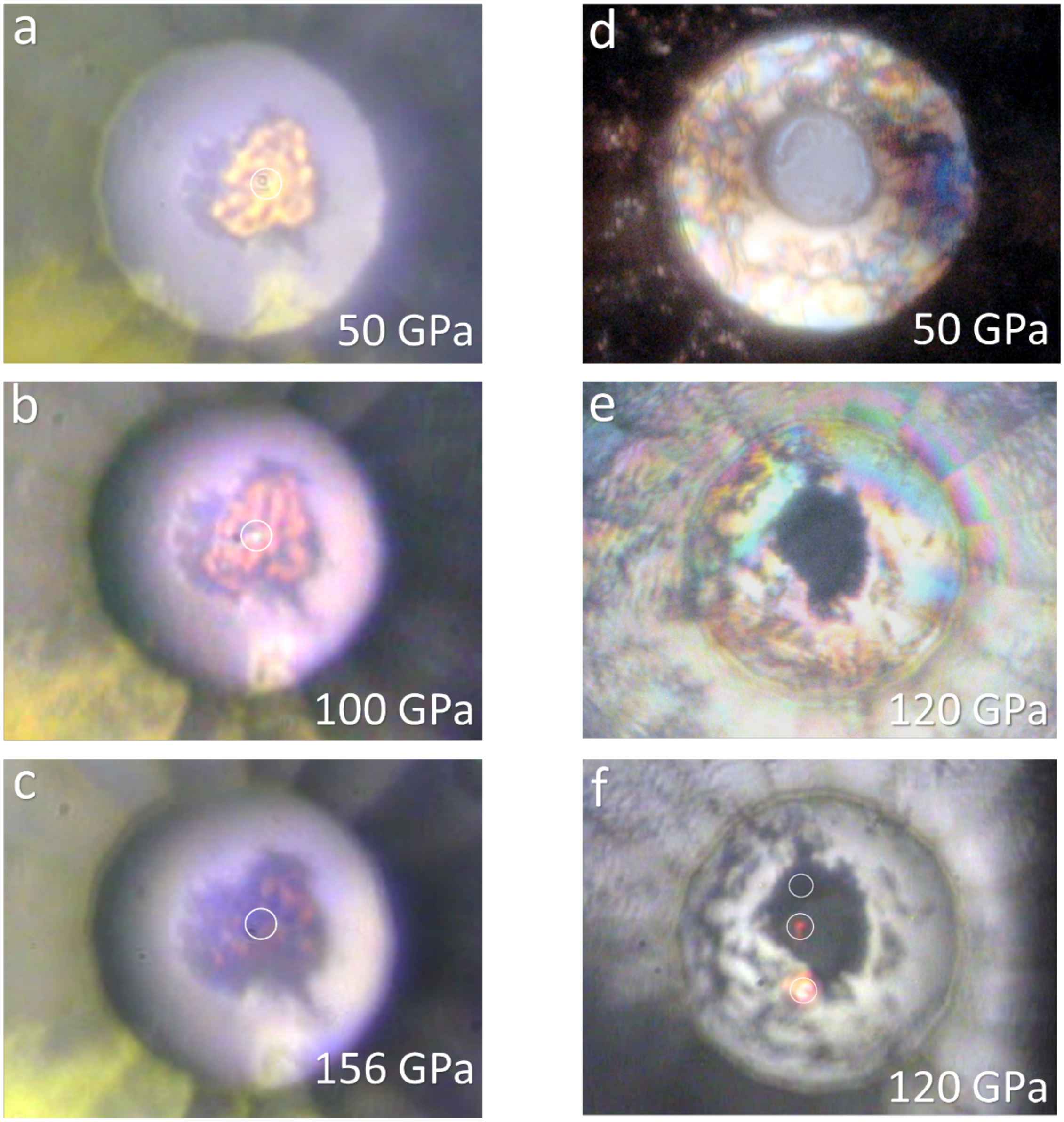

Figure S6. (a-c): The nitrogen-rich sample becomes less transparent to visible light on compression to 156 GPa. 514 nm laser light is incident in the centre of each sample indicated by white circles; (d-e); The Xe-rich sample was transparent at 50 GPa becoming opaque by 120 GPa; (e) Xe-rich sample at 120 GPa. 647 nm laser light of the same intensity is incident on the gasket and the sample as indicated by the white circles.

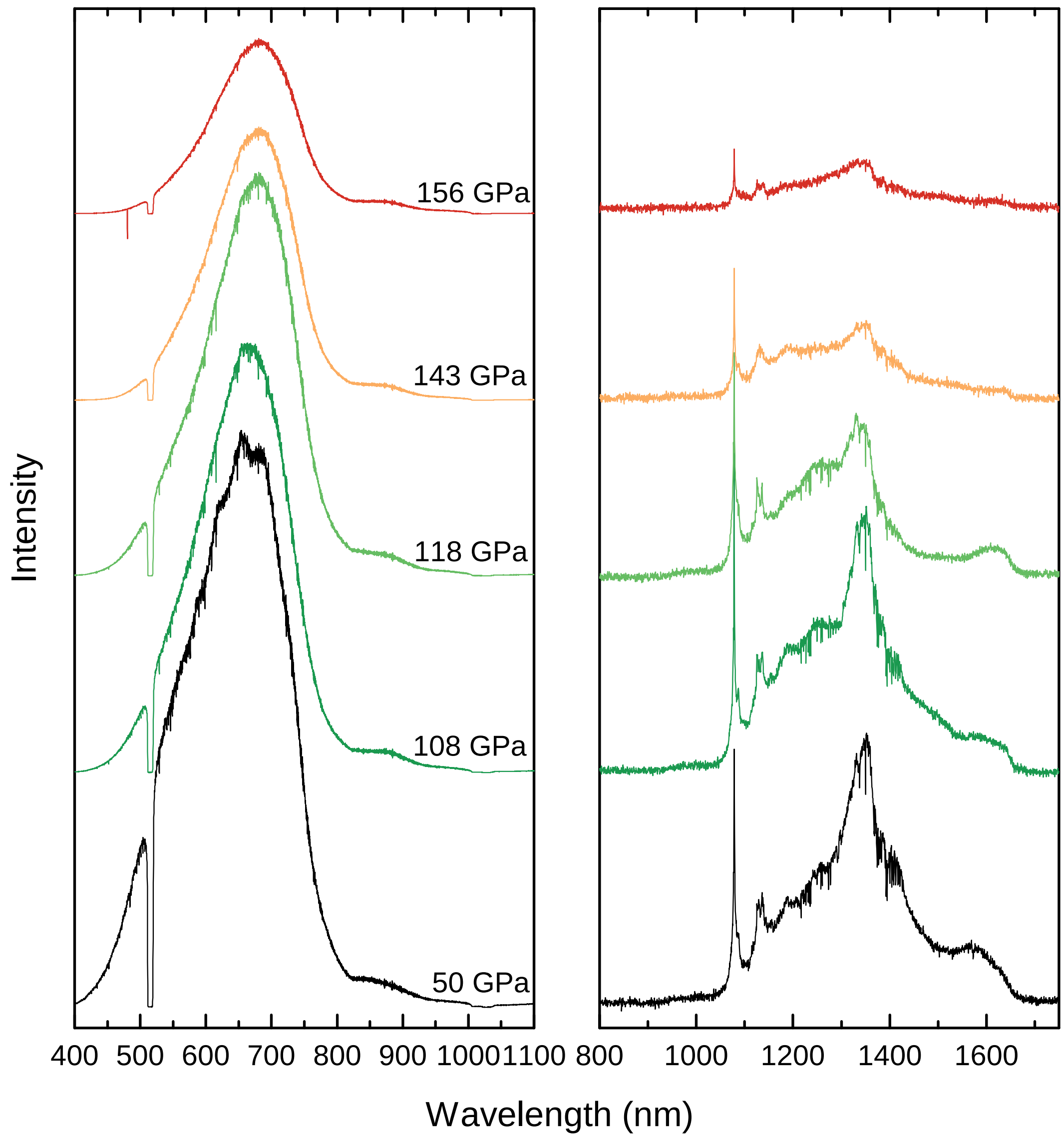

Figure S7. Raw transmission spectra for the N_2-rich sample in the visible (left) and mid infra-red regions (right) up to 156 GPa. The dip in intensity at 514 nm is due to the notch filters.
